# Do students’ personality traits change during medical training? A longitudinal cohort study

**DOI:** 10.1007/s10459-023-10205-2

**Published:** 2023-02-02

**Authors:** Milena Abbiati, Bernard Cerutti

**Affiliations:** 1 Unit of Development and Research in Medical Education (UDREM), University of Geneva, Medical School, 1 rue Michel Servet, 1211 Geneva, Switzerland; 2 Psychiatry Department, Unit of Forensic Psychiatry (IPL), Lausanne University Hospital, Lausanne, Switzerland

**Keywords:** Academic performance, Aptitude testing, Medical school selection, Personality testing

## Abstract

Many medical schools incorporate assessments of personal characteristics, including personality traits, in their selection process. However, little is known about whether changes in personality traits during medical training affect the predictive validity of personality assessments. The present study addressed this issue by examining the stability of personality traits and their predictive validity over a 6-year medical training course. Participants were two cohorts of Swiss medical students (N = 272, 72% of students admitted to Year 2) from whom we collected demographic data, Swiss medical studies aptitude test (EMS) scores, Big Five personality traits scores measured at three times and scores on the multiple-choice and objective structured clinical examination parts of the final medical examination. Our findings indicated that personality traits had medium-to-high rank-order stability (*r* > .60 over 3 years and *r* > .50 over 6 years). Mean-level changes were moderate for agreeableness (*d* = + 0.72) and small for neuroticism and conscientiousness (*d* = -0.29, *d* = -0.25, respectively). Individual reliable change indices ranged from 4.5% for openness to 23.8% for neuroticism. The predictive validity was similar to that of the first three years of follow-up. To the best of our knowledge, this is the first study to investigate changes in personality across undergraduate curriculum. Medical students’ personality traits were mostly stable across medical school and retain their predictive validity. Consequently, this study supports the use of tools measuring constructs underlying personality traits in selection. In addition, this study confirms that examination formats could favor students with certain personality traits.

Personality, which arises from an individual’s characteristic pattern of thinking, feeling, and behaving (Murthy et al., 2013), is a central concept in psychological research and a commonly used way of categorizing others. Informal evaluations such as “Jane is sweet” or “Joe is headstrong” fulfill people’s need to have coherent images of those around them and to be able to predict their reactions. In contrast, psychologists use objective measures of known validity and reliability to assess people’s personalities (Archer, 2014). Since the 1990s, most researchers have embraced the idea that there are five basic personality traits, known as the Big Five, namely, neuroticism, extraversion, openness to experience, conscientiousness, and agreeableness. Several tools have been developed to measure these traits and thereby quantify differences in people’s personalities (McCrae & Costa, 2003; Widiger, 2017). The NEO Five Factor Inventory (NEO-FFI) is the best known and most used of these tools.

Personality traits play both direct and mediating roles in medical students’ well-being, capacity for empathy, and career intentions (Eley et al., 2019; Haight et al., 2012; Hojat & Zuckerman, 2008; Lo et al., 2018; Mehmood et al., 2013; Mullola et al., 2019; Murthy et al., 2013; Prins et al., 2019; Toto et al., 2015). Moreover, four literature reviews conducted during the last two decades have found personality traits, especially extraversion and conscientiousness, to be the individual characteristics that most consistently associate with students’ academic performance (Chisholm-Burns et al., 2021; Doherty & Nugent, 2011; Ferguson et al., 2002; Hojat et al., 2013). This finding has led medical schools to use scores on personality traits associated with academic success, often measured indirectly via tools such as Multiple Mini Interviews and Situational Judgement Tests, as selection criteria for new students (Albanese et al., 2003; MacKenzie et al., 2017; Musson, 2009; Patterson et al., 2016; Powis, 2009; Powis et al., 2007).

Nevertheless, the research described above assumes that personality traits are stable characteristics (Caspi et al., 2005). This traditional view is now being challenged by research into maturational processes and recent theories according to which personality traits may vary as a result of psychosocial processes triggered by certain life situations and events (Ferguson & Lievens, 2017). One such event is the passage from school to university (Atherton et al., 2021; Bleidorn et al., 2018; Specht et al., 2011). The potential for people’s personalities to change has important implications for using personality evaluations as a selection criterion for medical students. Indeed, if students’ personalities evolve, pre-admission measures of personality traits may not correlate with future academic performance.

Two studies have investigated the predictive validity of personality assessments across medical training, but they both measured personality traits only on admission to medical school. Lievens and colleagues reported increases over time in the operational validity of extraversion, openness, and conscientiousness scores for predicting performance (Lievens et al., 2009), whereas Ferguson et al. found that high levels of conscientiousness were linked to better knowledge-based performance during preclinical training but to poorer clinical knowledge during clinical training. This result suggests that a trait can have both a “bright” side and a “dark” side (Ferguson et al., 2014).

The present longitudinal analysis is, to our knowledge, the first study to investigate changes in personality traits across initial medical training and to determine whether any such changes impact personality measures relation to examination performances. Changes in personality traits are most commonly assessed by determining their rank-order stability (consistency in the relative ordering of individuals on a given trait over time), mean-level changes (absolute mean-level change in scores for a given trait over time), and, to a lesser extent, individual changes (the magnitude of any change over time in an individual’s score on a trait) (Edmonds & Hill, 2020). To evaluate personality traits measures validity we used Messick’s unified validity framework, notably we sought evidence regarding relations to other variables (predictive validity) (Boateng et al., 2018; Cook & Beckman, 2006).

The present study used all of these methods to determine (1) whether personality traits change across medical school and (2) whether personality traits assessed at the beginning of medical school correlate with final examination performance, controlling for gender and examination format.

## Method

### Participants

Participants were students who entered Geneva Medical School in 2012 and 2013. To be included in the study, a student had to have completed the personality questionnaire at the beginning (Year 1) and end (Year 6) of medical school and to have taken the Swiss Federal Licensing Examination (FLE) at the end of Year 6.

### Data collection

The present study was part of a larger longitudinal research project conducted between 2011 and 2019 in which, each year, participants completed a questionnaire at the beginning of a compulsory class. The full method is described in previous papers (Abbiati et al., 2016; Piumatti et al., 2020). Before agreeing to participate in the study (by signing a consent form), students had been informed by email, ten days prior to signing session, of the content of the research project, their rights and commitments as volunteer participants, and the terms of confidentiality and privacy. The chair of the Cantonal Commission for Ethical Research exempted the present study from formal review.

Participants were asked to complete a personality questionnaire on three occasions during the 6-year medical training program: at the beginning of Year 1 (baseline), at the end of pre-clinical training (Year 3), and at the end of clinical training (Year 6). The measures of participants’ academic performance on entering and on finishing medical school were, respectively, their scores on the Swiss medical studies aptitude test (Eignungstest fur das Medizinstudium in der Schweiz, EMS) and on the FLE. These scores were provided by Geneva Faculty of Medicine and the Institute for Medical Education.

### Variables

#### Demographic data

Demographic data included in the study were age, gender, nationality (Swiss, European countries, Non-European countries), and type of high school diploma (scientific vs. other). We used parents’ level of education (primary, secondary, tertiary) as an indirect measure of a student’s socioeconomic level (UNESCO, 2011).

#### Personality

Participants completed the French version of the NEO Five Factor Inventory (NEO-FFI) (Aluja et al., 2005; Costa & McCrae, 1992), whose 60 items measure the Big Five personality traits (12 items per trait). Items are scored on 5-point Likert scales from 0 (strongly disagree) to 4 (strongly agree), giving a maximum score of 48. All five factors had acceptable internal reliability (Cronbach’s alphas were between 0.61 and 0.82), although extraversion and agreeableness had weaker internal reliability than neuroticism, openness to experience, and conscientiousness (Abbiati et al., 2014).

#### Performance on the EMS

As a measure of academic performance before medical school, we used participants’ scores on the EMS, which assesses students’ reasoning and problem-solving abilities, but not their scientific knowledge, communication skills, or social skills. Test scores are given as percentage rankings from 0 (low) to 100 (high). Medical schools in the German-speaking part of Switzerland use the EMS as an admissions test (Hänsgen & Spicher, 2011). Geneva University Medical School trialed the EMS between 2010 and 2012, during which period all applicants had to complete the test, although the results were not incorporated into the selection process (Cerutti et al., 2013).

#### Learning outcomes

Medical training at Geneva University Medical School is a 6-year program. At the time of the study students take a competitive examination at the end of Year 1 (average pass rate = 65%)(Abbiati et al., 2016) and a final written examination (the FLE) at the end of Year 6 (average pass rate = 99.5%). The FLE combines multiple-choice questions (MCQ) with objective structured clinical examinations (OSCE).

### Statistical analysis

We carried out supporting data and wave analyses to assess non-response biases and used attrition analysis to compare baseline personality measures for participants who did and who did not complete the personality measure at the end of medical school (Phillips et al., 2016). We then performed Pearson’s chi-squared tests to investigate dependence between the categorical variables and conducted analyses of variance to determine differences between groups of continuous variables.

We used Spearman correlation coefficients (*r*), Cohen’s *d* effect sizes, and Reliable Change Indices (RCI) to assess, respectively, personality rank-order stability, mean changes in personality traits between time-points, and changes in individuals’ personality traits (Christensen & Mendoza, 1986; Damian et al., 2019). Critical values were defined as following: for Spearman’s *r* : > 0.50 = high, 0.30 to 0.50 = moderate, and 0.25 to 0.30 = low; for Cohen’s d: > 0.80 = large, 0.30 to 0.80 = medium, and 0.20 to 0.30 = small (Cohen, 1988). We also used Pearson’s chi-squared tests to assess the reliable deviation from a stable situation (considered as follows: 2.5% of individuals with a decrease, 95% stable, and 2.5% with an increase).

We performed multiple linear regression analyses to determine whether MCQ and OSCE final examination scores correlate with personality traits, EMS, and gender. Results are given as percentages of variability explained (R^2^ and adjusted R^2^) and estimated coefficients with 95% confidence intervals.

Type I error rates were set at 0.05. All analyses were performed using R version 4.1.1 (R Foundation for Statistical Computing, Vienna, Austria) and SPSS version 24 (IBM Corp., Armonk, NY, USA).

## Results

### Main sample

Of the 419 students who completed the baseline personality questionnaire (78.5% response rate), 272 (156 females) were admitted to Year2. Mean age at Year 1 was 21 years (standard deviation 2.0). Gender and age distributions were similar to those for medical students within the Geneva area and within Switzerland (Office fédéral de statistique, 2021).

**Table 1:** Demographic Data and EMS Scores for the 419 Students Initially Enrolled in the Study

	All students	Students admitted to Year 2	Students not admitted to Year 2	P value	
	(N = 419)	(n = 272)	(n = 147)		
**Gender, n (%)**					
Female	266 (63.5%)	156^a^ (57.4%)	110^b^ (74.8%)	< 0.001	
Male	153 (36.5%)	116^a^ (42.6%)	37^b^ (25.2%)		
**Age, mean (SD)**					
Years	21.1 (2.0)	21.0 (2.1)	21.2 (1.7)	0.375	
**Nationality, n (%)**					
Swiss	333 (79.5%)	220^a^ (81.0%)	113^a^ (76.8%)		
European	79 (18.9%)	47^a^ (17.3%)	32^a^ (21.8%)	0.520	
Other	7 (1.6%)	5^a^ (1.7%)	2^a^ (1.4%)		
**Father’s education, n (%)**					
Primary	119 (28.5%)	80^a^ (24.1%)	39^b^ (36.8%)		
Secondary	97 (23.2%)	73^a^ (21.8%)	24^a^ (25.7%)	0.004	
Tertiary	203 (48.3%)	119^a^ (54.1%)	84^b^ (37.5%)		
**Mother’s education, n (%)**					
Primary	116 (27.7%)	60^a^ (22.3%)	56^b^ (37.9%)		
Secondary	135 (32.3%)	88^a^ (32.5%)	47^a^ (31.7%)	< 0.001	
Tertiary	168 (40.0%)	122^a^ (45.2%)	46^b^ (30.4%)		
**Type of high-school diploma, n (%)**					
Scientific	302 (72.1%)	199^a^ (73.1%)	103^a^ (70.4%)	0.576	
Other	117 (27.9%)	73^a^ (26.9%)	44^a^ (29.6%)		
**Medical studies aptitude test, mean (SD)**					
EMS	50.8 (10.9)	53.5 (10.7)	46.0 (9.6)	< 0.001	

For values expressed as n (%), the superscript letters a and b denote variables for which the proportions for students admitted to Year 2 and for students not admitted to Year 2 did not differ significantly at the 0.05 level

Table 1 compares students who did and who did not progress to Year 2 of medical school in 2012 and 2013. A wave analysis showed no differences in the two groups’ gender and age distributions. Most Year 1 students were Swiss, held a scientific high school diploma, and had college-educated parents. Males and students whose parents had had a college education were more likely to progress to Year 2 of medical school. Students who progressed (vs. did not progress) had higher EMS scores. All the students admitted to Year 2 sat the FLE at the end of Year 6 and therefore constituted our analytic sample for predictive validity.

### Personality traits longitudinal sample

Of the 272 students in our sample, 191 (69.2%) completed the personality questionnaire on all three occasions (Years 1, 3, and 6) and 53 (19.5%) completed the personality questionnaire in just Years 1 and 6. Applying the eligibility criteria (having completed the personality questionnaire in Year 1 and in Year 6 of medical school and to have taken the Swiss Federal Licensing Examination (FLE) at the end of Year 6) led us to exclude 28 participants (10.3%) from the personality traits stability analyses. Consequently 12 participants were excluded after the Year 1 data collection point; 16 participants were excluded after the Year 3 data collection point. There were no significant differences in personality traits between the included and the excluded students or between the 2012 and 2013 cohorts. As a result, our longitudinal personality traits sample (n = 244, 138 females) included 65% of the 376 students (226 females) who were admitted to Year 2 of medical school in 2012 or 2013, that is, 90% of the 272 students (156 females) who were admitted to Year 2 and who also completed the baseline questionnaire.

**Table 2:** Descriptive Statistics for the Big Five Personality Traits at Years 1 and 6

	All mean (SD) N = 244	Male mean (SD) n = 106	Female mean (SD) n = 138	p-value
Year 1				
Neuroticism	20.8 (8.6)	17.0 (8.0)	23.7 (7.9)	< 0.001
Extraversion	30.7 (5.3)	29.7 (5.3)	31.5 (5.3)	0.009
Openness	30.4 (6.1)	30.9 (6.0)	30.0 (6.2)	0.228
Agreeableness	29.4 (5.1)	28.0 (5.3)	30.5 (4.7)	< 0.001
Conscientiousness	35.3 (6.8)	34.1 (7.2)	36.3 (6.4)	0.014
Year 6				
Neuroticism	18.5 (7.6)	16.3 (7.6)	20.2 (7.2)	< 0.001
Extraversion	30.9 (5.9)	30.0 (6.6)	31.6 (5.3)	0.044
Openness	30.2 (6.2)	30.7 (6.0)	29.9 (6.3)	0.307
Agreeableness	33.0 (5.3)	31.1 (5.5)	34.4 (4.8)	< 0.001
Conscientiousness	33.9 (6.6)	32.7 (6.9)	34.8 (6.3)	0.017

Table 2 shows gender-stratified means and standard deviations for the five personality traits, measured in Year 1 and in Year 6. In both Years 1 and 6, females had higher scores than males for neuroticism and agreeableness. This was also the case, but to a lesser extent, for conscientiousness and extraversion.

### Rank-order stability

Table 3 shows personality rank-order stabilities (*r*) between Years 1 and 6, between Years 1 and 3, and between Years 3 and 6. Mean rank-order stability over six years ranged from 0.55 for extraversion to 0.62 for openness, with a mean of 0.56 for all five personality traits. Rank-order stabilities for most of the traits were above 0.50. The mean rank-order stabilities of the five personality traits were 0.68 for Years 1 to 3 and 0.69 for Years 3 to 6. Rank-order stabilities for most of the traits were above 0.60 for both 3-year periods.

**Table 3:** Test-Retest Correlations and Standardized Mean-Level Change Effect Sizes (Cohen’s d) for Each Personality Trait

	r (Cohen’s *d*)		
	Year 3-Year 1	Year 6-Year 3	Year 6-Year1
All	N = 207	N = 191	N = 244
Neuroticism	0.717 (-0.142)	0.666 (-0.209)	0.571 (-0.292)
Extraversion	0.650 (+ 0.155)	0.665 (-0.157)	0.552 (+ 0.037)
Openness	0.742 (-0.060)	0.785 (+ 0.019)	0.617 (-0.031)
Agreeableness	0.599 (+ 0.543)	0.692 (+ 0.239)	0.501 (+ 0.720)
Conscientiousness	0.669 (-0.344)	0.650 (-0.015)	0.581 (-0.250)
Mean	0.675	0.692	0.564

### Mean-level changes

Table 3 shows the Cohen’s *d* effect sizes for standardized mean-level changes in personality traits for males and females between Years 1 and 6, between Years 1 and 3, and between Years 3 and 6. Between Years 1 and 6 (mean ages 21.0 and 26.0 years, respectively), students’ mean agreeableness scores increased (*d* = + 0.72), whereas their mean neuroticism (*d* = -0.29) and conscientiousness (*d* = -0.25) scores decreased. Overall, changes in personality traits were greater between Years 1 and 3 than they were between Years 3 and 6: There was a moderate increase in agreeableness (*d* = + 0.56) and a moderate decrease in conscientiousness (*d* = -0.34) between Years 1 and 3, but only a small increase in agreeableness (*d* = + 0.24) and a small decrease in neuroticism (*d* = -0.21) between Years 3 and 6. Extraversion increased between Years 1 and 3 but decreased between Years 3 and 6 (*d* = + 0.30; *d* = -0.22, respectively).

### Individual changes

Reliable Change Indices (RCIs) showed no change in personality traits between Years 1 and 6 for between 74.2% and 95.5% of the students, depending on the trait considered (see Table 4). However, neuroticism, conscientiousness, extraversion, and agreeableness decreased significantly for 18.8%, 13.1%, 4.9%, and 1.20% of the students, respectively. Conversely, agreeableness, conscientiousness, neuroticism, and extraversion increased significantly for 14.3%, 7.4%, 7.0%, and 4.1% of the students. The only significant difference between gender was for neuroticism, which increased for 13.2% of males but for only 2.2% of females.

**Table 4:** Year-1 to Year-6 Reliable Change Indices for Individuals’ Personality Traits Stratified by Gender

	Decreased (%)		Stayed the same	Increased (%)	X^2^ (2, n = 244) differences between sex	X^2^ (2, n = 244) deviation from stability (2.5% 95% 2.5%)
Personality traits						
Neuroticism All		46 (18.8%)	181 (74.2%)	17 (7.0%)		291.6
Males		20 (18.9%)	72 (67.9%)	14 (13.2%)	11.5	p < .001
Females		26 (18.8%)	109 (79.0%)	3 (2.2%)	p = .003	
Extraversion All		12 (4.9%)	222 (91.0%)	10 (4.1%)		8.6
Males		4 (3.8%)	97 (91.5%)	5 (4.7%)	0.7	p = .013
Females		8 (5.8%)	125 (90.6%)	5 (3.6%)	p = .710	
Openness All		8 (3.3%)	233 (95.5%)	3 (1.2%)		2.2
Males		4 (3.8%)	100 (94.3%)	2 (1.9%)	0.8	p = .337
Females		4 (2.9%)	133 (96.4%)	1 (0.7%)	p = .660	
Agreeableness All		3 (1.2%)	206 (84.4%)	35 (14.3%)		141.4
Males		2 (1.9%)	88 (83.0%)	16 (15.1%)	0.8	p < .001
Females		1 (0.7%)	118 (85.5%)	19 (13.8%)	p = .680	
Conscientiousness All		32 (13.1%)	194 (79.5%)	18 (7.4%)		139.3
Males		15 (14.1%)	80 (79.5%)	11 (10.4%)	2.8	p < .001
Females		17 (12.3%)	114 (82.6%)	7 (5.1%)	p = .240	

Note: N = 488. Percentages for decrease, stay the same, and increase were based on Reliable Change Indices (RCI), where indices below − 1.96 or above 1.96 indicate reliable change. The RCIs used for this table were computed using two different standard errors of measurement derived from two separate sets of test–retest reliabilities (for long and short scales, respectively). We used chi-square tests to determine whether the actual distribution of non-changers and changers differed from the distribution if change were random (i.e., 95% stayed the same, 2.5% increased and 2.5% decreased)

### Multiple linear regression analysis

The linear regression analysis revealed links between FLE scores and EMS scores, personality traits, and gender (Table 5). EMS scores (MD = 0.25, p < .001) and conscientiousness (MD = 0.20, p = .029) correlated with higher FLE MCQ scores. Neuroticism (MD = 1.57, p < .001), extraversion (MD = 1.48, p = .024), being female (MD = -17.34, p = .025), and EMS scores (MD = 0.71, p = .025) correlated with higher FLE OSCE scores.

**Table 5:** Linear Regression Analysis of Gender, EMS Scores, and Personality Traits on Final Examination Performance

Variable	MD [95% CI]	P value	MD [95% CI]	P value
**Outcome**	FLE MCQ§		FLE OSCE°	
R^2^ Adjusted R^2^	0.1095 0.0851		0.1460 0.1227	
**Covariates**:				
Gender (male)	0.44 [-3.16; +2.29]	0.754	-17.34 [-32.46; -2.22]	0.025
EMS*	0.25 [+ 0.14; +0.36]	< 0.001	0.71 [+ 0.09; +1.32]	0.025
Neuroticism	0.14 [-0.02; +0.29]	0.096	1.57 [+ 0.68; +2.45]	< 0.001
Extraversion	0.10 [-0.13;0.34]	0.386	1.48 [+ 0.20; +2.77]	0.024
Openness	0.13 [-0.06; +0.32]	0.186	0.35 [-0.72; +1.42]	0.519
Agreeableness	-0.17 [-0.41; +0.07]	0.161	0.60 [-0.73; +1.92]	0.377
Conscientiousness	0.20 [+ 0.02; +0.38]	0.029	0.76[-0.24; +1.77]	0.134

*: Scores (0 to 100) are the number of marks gained expressed as a percentage of the total number of marks available

§: Standardized scores with a mean of 100 and a standard deviation of 10

°: Standardized scores with a mean of approximately 1200 and a standard deviation of 55

Note: MD = mean difference; CI = confidence interval; FLE = Final Licensing Examination; EMS = Swiss Medical Studies Aptitude Test.

## Discussion

Before deciding to integrate personality assessments into medical school selection processes, it is essential to determine whether students’ personalities change during the 6-year course. To this end, we conducted the first longitudinal study of rank-order stability, mean-level changes, and individual changes in the personality traits of two cohorts of medical students. We also analyzed the predictive validity of personality traits, EMS scores, and gender for performance on the MCQ and OSCE components of Switzerland’s medical licensing examination.

Results showed classic gender differences in personality traits and that students’ personality traits generally remained stable across medical school (Costa & McCrae, 1992). The personality traits rank-order stability correlations we obtained were similar to those reported by previous studies showing age-related changes in stability (e.g., rank-order correlations of between 0.40 and 0.50 at age 12 years and of around 0.70 at age 70 years, (Edmonds & Hill, 2020). Also in line with previous studies, the correlations we obtained decreased over the test-retest period (Costa & McCrae, 1992). Overall, the average mean-level change was just a quarter of a standard deviation and therefore too small to engender observable differences in everyday behavior (Hojat et al., 2013; Hojat & Zuckerman, 2008).

Individual-level analyses of personality traits revealed the proportion of students for whom changes in personality across the study period were reliable. These changes were similar to the mean-level changes, that is, they were mostly decreases in neuroticism and conscientiousness and increases in agreeableness. The changes in neuroticism and agreeableness were lower than but consistent with those reported by studies in developmental psychology (Borghuis et al., 2017; Costa et al., 2019; Damian et al., 2019; Hampson & Goldberg, 2020). This was not the case for the decrease in conscientiousness. Moreover, most of these changes occurred during preclinical training (Years 1 to 3), which suggests that they were due to a combination of students adapting to the new educational environment and their increasing maturity (Atherton et al., 2021; Specht et al., 2011). First-year medical students tend to be more conscientious than first-year psychology and science students, probably because students must be very conscientious, organized, efficient, and rational in order to successfully complete the highly competitive first year of medical school (Abbiati et al., 2016; Abbiati, Vaudraz, et al., 2015). However, they may get away with being less conscientious during the rest of their training, as drop-out rates are extremely low and the pass rate on the final examination is very high. Thus, the small decrease in conscientiousness during preclinical training suggests that some students are able to regulate this “bright” trait, within limits, according to their perceptions of situational requirements (Ferguson & Lievens, 2017). Similarly, students seem to regulate their neuroticism, a trait often perceived as “dark” due to its links with anxiety and depression, but which can also help people be more sensitive to emotions (Costa et al., 2019; Ferguson et al., 2014).

Finally, our results confirm the predictive validity of personality traits. In line with the findings of a preliminary 3-year study (Abbiati, Horcik, et al., 2015), conscientiousness and extraversion correlated significantly with examination performance, with only small differences in operational validity for both. Furthermore, although EMS scores correlated with OSCE and MCQ scores, gender and personality traits appear to be linked more strongly with OSCE performance than they are with MCQ performance. In other words, the enthusiasm, precision, and sensitivity to people’s feelings associated with higher levels of neuroticism and extraversion may be assets in examinations testing clinical skills, an aspect of medicine that cannot be tested using multiple-choice questions.

These insights into personality changes both in medical students as a group and in individual students are, nevertheless, subject to certain limitations. First, we explored the stability of personality in a medical school where students take a competitive examination at the end of Year 1 but face no further selection process. Although studies on different populations, in different cultures, and with different test-retest periods have reported similar findings regarding the overall stability of personality traits and the direction of any changes, our results may not be generalizable to settings involving selection processes at several stages during medical training (e.g., competitive final examination to enter certain residencies). Second, the self-report NEO-FFI questionnaire we used to assess personality traits is a reliable tool for measuring personality traits, but other methods, such as contextual measures and third-party evaluations, are also available. Finally, we assessed the stability of single personality traits (variable-centered approach), rather than the stability of overall personality profiles (person-centered approach).

## Conclusion

Medical students’ personality traits remain more-or-less stable across their training program and are usual correlates of examination performance that medical schools can incorporate into their student-selection process. Changes in some students’ personalities may be due, at least partly, to them adapting to their new learning context. Differences in the correlations between personality traits and different examination formats suggest that (medical) schools could adapt examination formats to favor certain personality traits.

Our results could help medical schools recruit students with a wider range of profiles. In fact, selection begins long before students apply to medical school, as some potential candidates may rule themselves out due to preconceived ideas about the types of student medical schools accept. Determining which traits students must have if they are to meet the medical profession’s and society’s changing needs and incorporating personality assessments into screening processes could enable medical schools to expand recruitment to students with different backgrounds and personal characteristics.
